# The Impact of College Matriculation Policies on the Cultural Adaptation of Migrant Children: A Statistical Analysis of Perceived Discrimination in Chinese Cities

**DOI:** 10.3390/bs15081136

**Published:** 2025-08-21

**Authors:** Xiaotong Zhi, Yun Sun, Zhendong Sun, Yuelong Ming, Cixian Lv

**Affiliations:** 1 School of Education Science, Qingdao University, Qingdao 266071, China; zhixiaotong@qdu.edu.cn (X.Z.); sunzhendong@qdu.edu.cn (Z.S.); 2 School of Education Science, Huazhong University of Science and Technology, Wuhan 430074, China; stnzunm@ucl.ac.uk; 3 Institute of Education, Xiamen University, Xiamen 361005, China

**Keywords:** college matriculation policy, migrant children, urban cultural adaptation, acculturation

## Abstract

Migrant children’s discrimination perceptions directly affect their cultural adaptation in the city of influx. In response to migrant children, cities in China have issued relevant urban education policies such as the different-location college entrance examination policy. This study aims to investigate the impact of China’s urban educational policies on the relationship between perceptions of discrimination and acculturation among migrant children. The research sample for this paper was drawn from nine cities that pioneered the policy reform, and a total of 1436 questionnaires were collected. This study analyzed the data using multiple regression analysis and mediation effect tests. This study reveals the following: (a) Migrant children’s educational policy identity has a significant positive impact on their acculturation, whereas their perception of discrimination has a significant negative effect on their acculturation. (b) As the influence of urban educational policies increases, the negative effects of discrimination perceptions on migrant children’s school cultural adaptation, community cultural adaptation, and customs and language adaptation will all diminish. To further explore the facilitating effect of urban educational policies on the cultural adaptation of migrant children, this study proposes recommendations for the household registration system, college entrance examination admission system, and child protection system. This paper not only puts forward policy recommendations for cities of inflow but also provides a Chinese research horizon for the urban cultural adaptation of migrant children in cities of inflow.

## 1. Introduction

An increasing body of empirical research has demonstrated that perceptions of discrimination significantly hinder the cultural adaptation of migrant children. For example, an empirical study by Zlobina et al. (2006) on children of international migrants found that discrimination perceptions had a significant positive predictive effect on urban cultural adaptation difficulties. Similarly, Chinese migrant children—who move from rural to urban areas—face comparable challenges in adapting to unfamiliar urban cultural environments. However, their adaptation process also exhibits unique characteristics, shaped by factors such as China’s household registration system, age characteristics, and the nature of their primary activity spaces (Ma & Wang, 2025).

While existing research has extensively examined the link between perceived discrimination and adaptation outcomes in international contexts, relatively little attention has been paid to domestic migrant children in China, especially concerning how educational policies might influence this relationship. Spencer et al. (2003a) suggested that social support can mitigate some of the negative effects of discrimination on adolescent development. In China, the introduction of relevant educational policies—particularly college matriculation policies—in the cities of inflow provides an important form of system-level support for the acculturation process of migrant children. However, the specific effects of such policies on mitigating discrimination-related adaptation difficulties remain underexplored.

To address this gap, the present study examines the relationship between college matriculation policy at different areas, perceived discrimination, and the urban cultural adaptation of migrant children in China. College matriculation policies in China, including secondary school and college entrance examination policies, have been gradually implemented across various provinces and cities. This study defines provinces that implemented policies early, have low entry barriers, and benefit from favorable external conditions as “pioneering breakthrough provinces”. The pioneering and experimental practices of these provinces in relevant fields provide important references and examples for other regions. In this case, in the period of China’s comprehensive deepening reform, what is the situation of discrimination perception and cultural adaptation of migrant children in these “breakthrough” provinces? Do college matriculation policies play a role in mitigating the relationship between perceptions of discrimination and acculturation among migrant children? What are the implications of the results for other provinces in sustaining the reform of education policies for migrant children?

By answering these questions, this study aims to contribute to both the theoretical understanding of migrant children’s acculturation and the practical development of inclusive educational policies. The findings are expected to inform ongoing reforms in China and provide valuable international reference points for other countries addressing similar challenges.

## 2. Theoretical Basis and Research Assumptions

### 2.1. Developmental History of College Matriculation Policies and Factors Influencing It

Generally speaking, urban cultural adaptation occurs through continuous and direct cultural contact between two culturally distinct groups. The process of acculturation involves two key aspects: the gradual abandonment of certain original cultural practices and the adoption of new cultural practices. The cumulative result of acculturation and countercultural experiences is an inherent shift towards assimilation into the dominant culture (Kim, 2017). It is evident that acculturation problems do not only exist between different races and nationalities. The problem of acculturation also exists between the two major groups of urban migrant workers and urban residents who possess different cultural types. Some scholars suggest that cultural adaptation includes psychological adaptation and socio-cultural adaptation (Gaitán-Aguilar et al., 2022; Searle & Ward, 1990). Psychological adaptation mainly refers to an individual’s psychological and physical well-being, including mental health, life satisfaction, and psychological well-being; cultural adaptation mainly refers to the level of an individual’s ability to learn social life skills, adapt to the local cultural environment, and interact effectively with members of the local group in a new cultural context, including behaviors, interpersonal communication, and linguistic adaptations (Ward & Kennedy, 1996). At first, researchers generally believed that the acculturation model was unidimensional, i.e., groups entering a new cultural environment would inevitably end up being assimilated into the dominant culture. The most typical representative theories are Park’s (1928) race relations cycle theory and Gordon’s (1964) seven-stage assimilation theory. With the depth of research, acculturation models began to shift from unidimensional to multidimensional. For example, most acculturation theories view acculturation as a complex micro- and macroprocess of ongoing contact between two different groups that follows a dynamic process (Bhatia & Ram, 2001, 2009). Berry’s (2003) four-fold model views acculturation as a developmental and interactive process that may result in perceptions at both the individual and group levels, as well as influences and behavioral changes in both receiving and immigrant societies. Considering that the policies adopted by the dominant group can also have an impact on immigrants, Bourhis et al. (1997) proposed a multidimensional model of acculturation, known as the interactive acculturation model. This model categorizes the acculturation process into four types, including assimilation, segregation, exclusion, and multiculturalism, based on the attitudes of the dominant group towards the non-dominant immigrant group. This multidimensional acculturation model pays attention not only to the subjective initiative of immigrants, but also to the public policies of the city of influx as well as the attitudes of members of the mainstream society. This dynamic and interactive perspective provides important insights for us to research the cultural adaptation of migrant children. 

Behaviorism suggests that when environmental stimuli change, individual psychology and behavior will change (Chang et al., 2024). So, what factors affect an individual’s acculturation? Research has found that two factors have a more significant impact on an individual’s adjustment status in a foreign culture, such as discrimination and prejudice (Zlobina et al., 2006), and group boundary permeability (Zhang et al., 2014). Zhang et al.’s study based on the urban and rural migrant population in China found that group boundary permeability was negatively correlated with prejudice in the urban and rural migrant population. Group boundary permeability (Permeability) first appeared in Tajfel and Turner’s (1986) social identity theory. The theory suggests that there are two main types of subjective belief systems in society: social mobility and social change. Beliefs about social mobility are based on resilient and permeable social structures. That is, individuals believe that the boundaries between groups are permeable and that individuals have the opportunity to move from one group to another. For example, a member of a disadvantaged group is accepted into a dominant group through further education, work, etc. The belief in social change, on the other hand, is based on a rigid social structure. That is, boundaries between groups are stable and impenetrable, and individuals are unable to leave their original group and enter the dominant group. However, when group boundaries are permeable, members of two dissimilar groups may be perceived as members of an integrated group, thus reducing intergroup prejudice (Dovidio et al., 2005). It is undeniable that Chinese College Matriculation Policy enhances the permeability of the boundary between the migrant children’s group and the dominant urban group. In addition to China, other countries have also enhanced the permeability of their borders through various policies. For example, India’s national education policy emphasizes multilingual education and inclusivity, aiming to promote social justice through education (Singh, 2025). In Germany, Welcome Classes or Additional Language Classes are provided for newly arrived immigrant children, typically lasting one year. These programs are designed to help immigrant children better adapt to the mainstream school system in Germany through targeted language training (Hanover et al., 2020). Therefore, the policy is actually a positive signal that conveys to migrant children the belief that “as long as I work hard, I will have a chance to enter another group”. This makes them more optimistic and positive about the future.

In this study, we adopt the Interactive Acculturation Model proposed by Bourhis et al. (1997) as our primary theoretical framework. This model is particularly relevant to our research because it provides a comprehensive understanding of how different acculturation strategies influence adaptation outcomes in multicultural contexts. In addition, it explicitly considers the interactive nature of acculturation processes between dominant and non-dominant groups, which is crucial for our study focusing on urban cultural adaptation.

### 2.2. Relationship Between Discrimination and Urban Cultural Adaptation

There is a very different cultural context between the urban and the rural areas, for example, in terms of the way society is organized, the degree of social differentiation, and the way of life. Therefore, the movement of migrant children to the city is not only a geographic move, but also an “acculturation” in the modern sense of the word. For example, Berry and Sabatier (2010) argue that internal migrants face the same acculturation problems as international migrants. The acculturation problem of immigrants can be examined from different dimensions; Phinney (1990) argues that acculturation can be divided into five dimensions, including cultural activities, cultural values, ethnic identity, interpersonal relationships, and language preferences, etc. Ying (1995) examined 143 Chinese Americans, and argued that linguistic orientation, acculturation to cultural activities, and acculturation to social relationships are important dimensions. Language orientation is the prerequisite and tool, which is the most important element of cultural adaptation (Buchanan et al., 2018); cultural activity adaptation is mainly the degree of acceptance and participation in customs and traditions, cultural traditions, etc.; and social relationship adaptation mainly refers to the interactions and interactions with members of the urban mainstream. The cultural adaptation problems of domestic migrant children have certain specificities, for example, caused by factors such as household registration system, age characteristics, and main activity venues (Cao et al., 2024). Therefore, we categorize the cultural adaptation of migrant children into three dimensions: school cultural adaptation, community cultural adaptation, and urban customs and dialect adaptation.

Discrimination is a complex social phenomenon. Most of the discriminatory behaviors in society are manifested in subtle ways that are difficult to measure objectively. Therefore, researchers at home and abroad have gradually turned their attention to the fact that the research object can perceive discrimination in the daily field, i.e., perception of discrimination. Perception of discrimination is a subjective experience directly related to group membership as opposed to objective discrimination. Perception of discrimination has been defined as “the subjectively perceived consequences of discrimination one faces in everyday experience” (Schmitt et al., 2014). Discrimination perceptions include both the perception of discrimination directed towards oneself and the perception of discrimination directed towards one’s own group (Liu & Zhao, 2016). The latter can be called group discrimination perception, which is one of the important variables in this paper.

So, how does discrimination perception affect the urban cultural adaptation of migrant children? The study of Xiong et al. (2021) proved that there is a significant positive correlation between the individual’s perception of social support and the pro-social tendency of migrant children. A large number of scholars have verified the relationship between discrimination perceptions and acculturation in various types of mobile populations. The results found that discrimination perceptions of migrant populations are negatively related to acculturation (Yang et al., 2023; Félix, 2006). In addition, the phenomenological variant theory of ecological systems theory describes the social cognitive development process of adolescents in disadvantaged groups. Based on this theory, the present study argues that if vulnerable groups are not provided with appropriate social support resources, and consequently do not develop appropriate coping strategies, then discrimination acts as a risk factor that directly produces unfavorable consequences (Spencer et al., 2003a). This implies that there is a causal relationship between discrimination perceptions and the degree of social adaptation, which plays an initiating role in the adaptation process.

Based on the above research, the following hypotheses are proposed in this study:

**H1a.**  *Discrimination perception has a significant negative effect on migrant children’s school culture adaptation.*

**H1b.**  *Discrimination perceptions have a significant negative effect on migrant children’s community cultural adaptation.*

**H1c.**  *Discrimination perceptions have a significant negative effect on migrant children’s customs and language adaptation.*

### 2.3. Relationship Between the College Matriculation Policy and Discrimination Perception and Acculturation

In fact, not all migrant children experiencing discrimination will have an adverse effect on their acculturation. Does this mean that there are other influencing factors between discrimination perception and acculturation? Studies at home and abroad have shown that group boundary permeability affects the cultural adaptation of group members (Yang et al., 2023). In other words, under the condition of boundary permeability, members of disadvantaged groups will show lower in-group preference and less out-group discrimination, which contributes to the cultural adaptation of disadvantaged groups (Ellemers et al., 2010). For example, Zhang et al. (2014) found that the permeability of group boundaries is negatively correlated with the biases against urban and rural migrant populations. This permeability enhances the sense of identity of vulnerable group members towards mainstream culture, thereby reducing psychological pressure and cultural conflicts caused by group differences (Ren et al., 2023).

Clearly, the introduction of the college matriculation policies can increase the permeability of group boundaries.

Thus, this study introduces the variable of college matriculation policies into the study of acculturation of migrant children and proposes the following hypothesis:

**H2a.**  *College matriculation policies have a significant positive effect on migrant children’s cultural adaptation on campus.*

**H2b.**  *College matriculation policies have a significant positive effect on the community cultural adaptation of migrant children.*

**H2c.**  *College matriculation policies have a significant positive effect on migrant children’s adaptation to customs and language.*

In addition, research has shown that social support also plays an important moderating role between perceived discrimination and cultural adaptation. For example, Mutluhan and Mustafa (2025) found that a decrease in social support partially explains the threatening effect of perceived discrimination on socio-cultural adaptation. This suggests that when migrant children feel more social support, their perception of discrimination may decrease, thereby facilitating their cultural adaptation. Spencer et al. (2003a) also emphasized the importance of social support in mitigating the adverse effects of discrimination on adolescent development. The college matriculation policy, as a social policy that increases the permeability of group boundaries, can also provide good social support for migrant children. This implies that the policy may influence both discrimination perceptions and migrant children’s acculturation.

We theorize that perceived discrimination and policy perception significantly influence adaptation outcomes through their impact on individuals’ psychological well-being and social integration. Specifically, we hypothesize that higher levels of perceived discrimination will lead to poorer adaptation outcomes, while positive policy perception will facilitate better adaptation. This relationship is supported by previous research indicating that perceived discrimination can undermine psychological well-being and social cohesion (Schmitt et al., 2014), while supportive policies can enhance social integration and cultural adaptation (Lee et al., 2004). Based on this, this study proposes the following hypotheses:

**H3a.**  *College matriculation policies play a negative moderating role in the relationship between perceived discrimination and school cultural adaptation of migrant children.*

**H3b.**  *College matriculation policies play a negative moderating role in the relationship between migrant children’s perceived discrimination and community cultural adaptation.*

**H3c.**  *College matriculation policies play a negative moderating role in the relationship between migrant children’s perceived discrimination and their adaptation to customs and language.*

## 3. Research Design, Materials, and Methods

### 3.1. Study Sample and Data Collection

This study used stratified whole-cluster sampling to randomly select school students from a number of cities for questionnaire and field research. These cities are all from provinces that implemented the college matriculation policies earlier. Moreover, the distribution of migrant children in these cities is more concentrated, such as Hangzhou, Ningbo, Wenzhou, Fuzhou, Shijiazhuang, Jinan, Nanchang, Hefei, Wuhan, Changsha, and Zhengzhou. They come from the following provinces: Zhejiang, Fujian, Hebei, Shandong, Jiangxi, Anhui, Hubei, Hunan, and Henan. Considering the instability of migrant children’s migration and the fact that it takes a process for them to perceive the college matriculation policies, the target respondents were selected to be school students in grades 8 to 12. Moreover, the target schools were all urban public schools where the proportion of migrant children accounted for more than 15 per cent of the total number of students in the school. This study was approved by the school’s ethical committee, and all subject groups filled out the questionnaires under the guidance of their guardians, and informed consent was obtained from both the subject groups and their guardians. Eventually, 1436 valid questionnaires were recovered from this survey, with a recovery validity rate of 78%. The descriptive statistics of the questionnaire are shown in Table 1.

Table 1 shows that girls account for a relatively large proportion of the students surveyed, at 62.1 percent. In terms of household mobility, there are about four times as many students moving from “village to town” as from “town to town”, which is roughly in line with the ratio of the two categories at the national level. In terms of the composition of the population in their place of residence, the majority of students, 58.5 per cent, are surrounded by local people. In the survey of fathers’ occupations, the vast majority of students’ fathers are general laborers, self-employed, general managers, and general technicians, with a small proportion of middle managers. In the survey on the education level of students’ fathers, the vast majority of students’ fathers had a high school education level, accounting for 56.3 per cent; 39.3 per cent had a middle school education level; and 2.6 per cent had a bachelor’s degree.

### 3.2. Variable Definition and Measurement

#### 3.2.1. Demographic Variables

This mainly refers to those control variables that may affect the dependent variable or interfere with the relationship between the main independent variable and the mediator variable. Based on the results of existing research at Chinese and international level, and based on the accessibility of the data, the demographic variables in this study include gender, type of household mobility, structure of people in the place of residence, father’s education level, and father’s occupation type.

#### 3.2.2. Cultural Adaptation Questionnaire

This questionnaire is mainly used to measure the ability of cross-cultural migrants to adapt in a new culture and is mainly based on Ward and Kennedy’s (1999) acculturation scale. The scale is divided into two main dimensions, the first reflecting the migrants’ knowledge of the new culture and their ability to communicate with locals, and the second focusing on interpersonal interactions and self-management of awkward situations. Since this study focuses on Chinese migrant children in middle and high school, the following modifications were made to the scale: first, the indicators related to adults in the scale were removed, such as “coping with academic work” and “dealing with governmental agencies.” Firstly, indicators relating to adults were removed from the scale, such as “coping with academic work”, “dealing with government agencies”, and “dealing with authority figures”. Secondly, indicators related to emigration, ethnicity, and race were removed, such as “living abroad without family or living alone without parents,” “dealing with people abroad in the university,” “worshipping as usual”, “accepting or understanding the local political system”, and “interacting with people of different races”.

Finally, 18 indicators in 3 aspects were formed according to the particularities of the migrant children group. The first aspect is adaptation to urban school culture, including six items such as “making friends with local classmates” and “understanding what they need to learn at school”; the second aspect is adaptation to urban community culture, including six items such as “ Participating in community cultural activities and gatherings”, “Adapting to the local climate”, “Adapting to local accommodation”, and six other items; the third aspect is adaptation to urban customs and dialects, including “Adapting to the local climate”, “adapting to local accommodation”, and six other articles. The third aspect is adaptation to urban customs and dialects, including six items such as “getting used to local food” and “being able to understand the accent or dialect of local people”. The questionnaire consists of 41 questions on a 5-point Likert scale from 1 to 5 indicating the degree of adaptation compared to the respondent’s actual situation, with 1 indicating “very unadapted” and 5 indicating “very well adapted”, and the higher the score, the better the adaptation, and vice versa. The higher the score, the better the adaptation, and vice versa. The higher the score, the better the level of acculturation of the respondent and vice versa.

#### 3.2.3. Perceived Discrimination Questionnaire

This questionnaire examines the extent to which migrant children subjectively feel discriminated against or treated unfairly because of their non-residential status. The questionnaire mainly refers to the “Discrimination Perception Questionnaire” developed by Krahé et al. (2005). According to the special characteristics of the migrant children group, the questionnaire finally formed 12 indicators in three areas. First, perceived discrimination from schools, teachers, and classmates, with 4 items, such as “Compared with local students, we migrant children are often criticised more harshly when we make the same mistakes”; second, perceived discrimination from community residents and other groups, with 4 items, such as “I often hear that local people are discriminatory or mean to us”; thirdly, perceived discrimination from various systems and policies, with 4 items, such as “The education policy of this city does not protect our rights and interests”.

The questionnaire also used a five-point Likert scoring method, with higher scores indicating more serious perceptions of discrimination among the respondents.

#### 3.2.4. The Questionnaire on the College Matriculation Policies

While college matriculation policies in each province vary across provinces and objectively affect each individual in a different way and to a different extent, they are difficult to measure directly. Therefore, drawing upon previous measures of social support, policy perception, and educational equity, we focus on capturing migrant children’s perceptions of the college matriculation policies (Aguinis et al., 2021; Dong et al., 2025).

Based on the educational backgrounds and actual needs of migrant children, a preliminary item pool covering multiple dimensions was generated. In order to ensure the scientific rigor and applicability of the scale, we consulted experts in the fields of education, psychology, and sociology following multiple rounds of field surveys conducted in various cities. Experts evaluated the preliminary generated entries and provided suggestions for modification. After multiple rounds of expert consultation, nine terms were finally selected, forming a three-dimensional scale. The first dimension, captures cognitive understanding of the policy, such as “After the introduction of the College Matriculation Policy, my family and I have a better understanding of the policy”. The second dimension reflects migrant children’s perceptions of policy fairness, such as “Most of the migrant around me children around me think that the local college matriculation policies are fair”. And the third dimension measures the perceived impact of the local college matriculation policies on migrant children, e.g., “The introduction of the college matriculation policies has made it possible for me to study in the city with more peace of mind”. The questionnaire used a five-point Likert scale, with higher scores indicating better perceptions of the college matriculation policies, and vice versa.

### 3.3. Reliability and Validity Tests

#### 3.3.1. Reliability Analysis

In this study, CITC analysis and Cronbach’s alpha coefficient method were used to test the reliability of the measurement terms and to purify the measurement terms. According to the criteria of CITC greater than 0.5 and Cronbach’s alpha coefficient greater than 0.7, all measurement terms in this study met the reliability test.

As shown in Table 2, the total Cronbach’s alpha values for Perceived Discrimination, College Matriculation Policy, and Cultural Adaptation are 0.938, 0.955, and 0.956, all above 0.7. The CITC values for all items are above 0.5, and the Cronbach’s alpha values for the three dimensions of Cultural Adaptation are 0.936, 0.920, and 0.923, all above 0.7, indicating good reliability.

#### 3.3.2. Validity Analysis

The Perceived Discrimination and Cultural Adaptation scales are based on well-established theories, while the College Matriculation Policy scale was developed through extensive research and revision. Thus, all three scales have good content validity. To test structural validity, exploratory factor analysis and confirmatory factor analysis were used. Results of exploratory factor analysis are shown in Table 3. The KMO values for perceived discrimination, college matriculation policy, and cultural adaptation are greater than 0.9, and all passed Bartlett’s test. Perceived discrimination and college matriculation policy each extracted one common factor, while cultural adaptation extracted three factors, with cumulative variance explanations all above 60%. Therefore, the exploratory factor analysis results are consistent with the assumptions of this study.

Next, a confirmatory factor analysis was conducted. Structural equation models were built separately for perceived discrimination, college matriculation policy, and urban cultural adaptation; the model-fit results are shown in Table 4. For all three models, Prob > chi^2^ = 0.000 < 0.05; CD, CFI, and TLI are all close to 0.9; and RMSEA is below 0.1 (close to 0.1), indicating that each model fits well overall. Moreover, all standardized factor loadings exceed 0.7, and every estimated parameter in the models reaches statistical significance, demonstrating strong internal quality. The t-text results show that the *p*-value for each parameter is greater than the significance level, and all item parameters are significant. Therefore, the scale used in this study exhibits good construct validity.

#### 3.3.3. Common Method Bias Test

To address potential common method bias arising from factors such as sample characteristics and survey timing, this study implemented procedural remedies, for example, dispersing items measuring the same construct across the questionnaire layout. Harman’s single test was applied by subjecting all measurement items to an unrotated factor analysis. If only one factor emerges or if the first factor accounts for the vast majority of variance, serious common method bias is indicated; otherwise, it is not. The results showed no single dominant factor, and the first principal component explained 41.354% of the total variance, below the 50% threshold, indicating that common method bias is not a concern in these data.

### 3.4. Analysis Method

This study conducted reliability and validity tests on each measurement item. The results indicate that the scales used in this research exhibit good reliability and validity. In addition, analysis found no common method bias in the data. First, a variance analysis was performed to further explore the specific distinctions among different categories of migrant children. Then, the research hypotheses were tested through correlation and regression analyses, examining the relationships among variables such as policy perception, policy perception, perceived discrimination, and urban cultural adaptation.

## 4. Research Results

### 4.1. Variance Analysis of the Migrant Children Group

Previous research has shown considerable heterogeneity within the migrant children population. To further explore specific differences among subgroups of migrant children, an independent sample test or one-way analysis of variance were conducted for each control variable. The results are presented in Table 5 and Table 6:

Regarding gender, migrant children’s perceived discrimination and community cultural adaptation, including adaptation to local customs and language, showed no significant differences; significant differences were found in college matriculation policy and campus cultural adaptation. Specifically, male migrant children have a significantly lower perception of college matriculation policy than females (t = −8.343, *p* < 0.05), while female migrant children have a significantly higher perception of campus culture adaptation than males (t = −11.582, *p* < 0.01).

Regarding the type of household mobility, significant differences were observed for migrant children’s perceived discrimination, college matriculation policy, campus cultural adaptation, community cultural adaptation, and adaptation to local customs and language. Children in the “rural-urban” migration category exhibited significantly higher perceived discrimination than those in the “urban-urban” category; by contrast, the “urban-urban” group demonstrated significantly higher scores on college matriculation policy and cultural adaptation compared to the “rural-urban” group.

With respect to the structure of people in the place of residence, migrant children’s perceived discrimination, college matriculation policy, and urban cultural adaptation all showed significant differences. Multiple comparisons indicated that migrant children living in areas with predominantly local residents reported significantly lower perceived discrimination than those in areas with predominantly non-local or mixed populations; those in predominantly local areas also demonstrated significantly greater familiarity with the college matriculation policy and perceived its fairness more highly than their counterparts in predominantly non-local or mixed areas; and migrant children residing among mostly local residents exhibited significantly higher levels of urban cultural adaptation than those living in predominantly non-local or mixed communities.

In terms of father’s education level, migrant children’s perceived discrimination, college matriculation policy, and urban cultural adaptation all exhibited significant differences. Multiple comparisons indicated that migrant children whose fathers have a bachelor’s degree or above display significantly lower perceived discrimination levels than those whose fathers have only a junior school or high school education; the higher the father’s education level, the greater migrant children’s familiarity with and perceived fairness of the college matriculation policy; and migrant children whose fathers have a bachelor’s degree or above demonstrate significantly higher urban cultural adaptation levels than those whose fathers have a high school education or below.

Regarding father’s occupation type, migrant children’s perceived discrimination, college matriculation policy, and urban cultural adaptation all showed significant differences. Post hoc multiple comparisons revealed that children whose father’s occupation was senior management, senior technical staff, or private business owner exhibited the lowest levels of perceived discrimination, the highest familiarity with and perceived fairness of the college matriculation policy, and the highest levels of urban cultural adaptation; children whose father’s occupation was general management or technical personnel showed above average levels of campus cultural adaptation, overall urban cultural adaptation, adaptation to local customs, and language adaptation.

### 4.2. Correlation Analysis

The results of the correlation analysis are shown in Table 7.

Perceived discrimination was significantly negatively correlated with campus cultural adaptation (r = −0.428, *p* < 0.01), community cultural adaptation (r = −0.372, *p* < 0.01), and customs and language adaptation (r = −0.387, *p* < 0.01). Perceived discrimination was also significantly negatively correlated with the acceptance of college matriculation policy (r = −0.382, *p* < 0.01). The college matriculation policy acceptance was significantly positively correlated with campus cultural adaptation (r = 0.717, *p* < 0.01), community cultural adaptation (r = 0.814, *p* < 0.01), and adaptation to local customs and language (r = 0.786, *p* < 0.01). Some hypotheses received preliminary support, but further regression analysis is required for validation.

### 4.3. Test of Moderating Effects

This study employed hierarchical linear regression models. To distinguish the effects of control variables, independent variables, moderator variables, and their interaction terms, variables were entered into the regression models stepwise. To avoid potential multicollinearity arising from including interaction terms, independent variables and moderator variables were mean-centered before their interaction terms were computed and added to the models. In all models, the runs test *p* values exceeded 0.05, indicating independence of residuals; variance inflation factors (VIFs) ranged from 1 to 10, showing no significant multicollinearity; and data in the P-P plots fell between –2 and 2, demonstrating homoscedasticity.

In Table 8, the results present the moderating effect of the college matriculation policy on the relationship between perceived discrimination and campus cultural adaptation. Model 1 tested the regression of control variables on campus cultural adaptation: gender negatively predicted campus cultural adaptation (β = −0.228, *p* < 0.01); type of household mobility positively predicted campus cultural adaptation (β = 0.215, *p* < 0.01); structure of people in the place of residence positively predicted campus cultural adaptation (β = 0.369, *p* < 0.01); father’s education level positively predicted campus cultural adaptation (β = 0.171, *p* < 0.01); and father’s occupation type positively predicted campus cultural adaptation (β = 0.220, *p* < 0.01).

In Model 2, after controlling for variables such as gender and type of household mobility, perceived discrimination significantly negatively predicted campus cultural adaptation (β = −0.16, *p* < 0.01), supporting hypothesisH1a.

In Model 3, after controlling for variables such as gender and type of household mobility, college matriculation policy significantly positively predicted campus cultural adaptation (β = 0.375, *p* < 0.01), supporting hypothesis H2a.

Model 4 builds on Model 3 by including an interaction term between perceived discrimination and college matriculation policy, which increases the adjusted *R*^2^ from 67.5% to 77.2%. The regression coefficient for the interaction term on campus cultural adaptation is 0.475 (*p* < 0.01), indicating that college matriculation policy moderates the relationship. The main effect of perceived discrimination on campus cultural adaptation is negative (β = −0.097), while the interaction term’s coefficient is positive. After adding the interaction, the coefficient for perceived discrimination changes to β = −0.030 in absolute value, showing a reduction. This indicates that college matriculation policy attenuates the negative predictive effect of perceived discrimination on campus cultural adaptation. Therefore, as the influence of college matriculation policy increases, the adverse impact of perceived discrimination on campus cultural adaptation weakens. Hypothesis H3a is supported.

In Table 9, the results display the moderation test of college matriculation policy on the relationship between perceived discrimination and community cultural adaptation. Model 5 presents the regression analysis of control variables predicting community cultural adaptation. Gender emerged as a significant negative predictor (β = −0.176, *p* < 0.01), type of household mobility as a significant positive predictor (β = 0.142, *p* < 0.01), structure of people in the place of residence as a significant positive predictor (β = 0.252, *p* < 0.01), father’s education level as a significant positive predictor (β = 0.131, *p* < 0.05), and father’s occupation type as a significant positive predictor (β = 0.313, *p* < 0.01).

Model 6, after controlling for gender, type of household mobility, and other covariates, shows that perceived discrimination is a significant negative predictor of community cultural adaptation (β = −0.143, *p* < 0.01), confirming hypothesis H1b.

Model 7, after controlling for gender, type of household mobility, and other covariates, shows that college matriculation policy is a significant positive predictor of community cultural adaptation (β = 0.661, *p* < 0.01), confirming hypothesis H2b. However, in Model 7, we introduced “discrimination perception” and “College Matriculation Policy Acceptance” as new predictor variables. Compared with Model 5, the significance of some control variables has changed. Specifically, the type of household mobility (Model 5: β = 0.142, *p* = 0.004; Model 7: β = 0.028, *p* = 0.443), structure of people in the place of residence (Model 5: β = 0.252, *p* = 0.000; Model 7: β = 0.072, *p* = 0.098), and father’s education level (Model 5: β = 0.131, *p* = 0.010; Model 7: β = 0.053, *p* = 0.158) are no longer significant in Model 7. This may be due to collinearity between the newly introduced variables and these control variables, or because the addition of new variables altered the explanatory structure of the model, thereby affecting the significance of these control variables.

Model 8 adds an interaction term between perceived discrimination and college matriculation policy to Model 7, raising the adjusted *R*^2^ from 69.8 percent to 70.4 percent. The regression coefficient for the interaction term on community cultural adaptation is 0.132, *p* < 0.05, indicating that college matriculation policy moderates their relationship. Because the coefficient of perceived discrimination on community cultural adaptation is negative while the interaction term’s coefficient is positive, and after including the interaction term the coefficient of perceived discrimination changes from −0.032 to −0.024 and its absolute value decreases, college matriculation policy weakens the negative predictive effect of perceived discrimination on community cultural adaptation. Therefore, as the influence of college matriculation policy increases, the negative impact of perceived discrimination on community cultural adaptation diminishes. Hypothesis H3b is supported.

In Table 10, the results present the test of the moderating effect of college matriculation policy on the relationship between perceived discrimination and customs and language adaptation. Model 9 reports the regression of control variables on customs and language adaptation. Gender negatively and significantly predicts adaptation to customs and language adaptation, β = −0.153, *p* < 0.01; type of household mobility positively and significantly predicts adaptation to customs and language adaptation, β = 0.114, *p* < 0.05; structure of people in the place of residence positively and significantly predicts adaptation to customs and language adaptation β = 0.352, *p* < 0.01; father’s educational level positively and significantly predicts adaptation to customs and language adaptation, β = 0.123, *p* < 0.05; and father’s occupation type positively and significantly predicts adaptation to customs and language adaptation, β = 0.283, *p* < 0.01.

Model 10, with gender, type of household mobility, and other variables controlled, shows perceived discrimination to be a significant negative predictor of customs and language adaptation (β = −0.146, *p* < 0.01), supporting H1c.

Model 11, with gender, type of household mobility and other variables controlled, shows college matriculation policy to be a significant positive predictor of customs and language adaptation (β = 0.581, *p* < 0.01), supporting H2c.

Model 12 builds on Model 11 by adding the interaction term between perceived discrimination and college matriculation policy. Adjusted R-squared increases from 68.6 percent to 69 percent. The interaction term’s regression coefficient on customs and language adaptation is 0.104, *p* < 0.05, indicating that college matriculation policy moderates their relationship. Since perceived discrimination’s coefficient on customs and language adaptation is negative while the interaction term’s coefficient is positive, and because perceived discrimination’s coefficient shifts from −0.049 to −0.022, absolute value decreases, after adding the interaction, college matriculation policy attenuates the negative predictive effect of perceived discrimination on customs and language adaptation. Therefore, as the influence of college matriculation policy increases, the negative impact of perceived discrimination on customs and language adaptation weakens. H3c is supported.

## 5. Discussion

This study provides robust empirical evidence regarding the dynamic relationship between perceived discrimination, identification with college matriculation policy, and urban cultural adaptation among migrant children. The findings elucidate both the risk and protective factors influencing migrant children’s adaptation outcomes in destination cities.

First, consistent with the extant literature, this study confirms that perceived discrimination exerts a significant negative impact on migrant children’s urban cultural adaptation, across the dimensions of campus cultural adaptation, community cultural adaptation, and customs and language adaptation (Buchanan et al., 2018; Ward & Kennedy, 1999; Spencer et al., 2003b). The negative association identified aligns with prior findings that perceived discrimination acts as a salient risk factor for vulnerable migrant populations, impeding their psychosocial well-being and cultural adjustment (Brownfield & Thompson, 2005; Xu & Lv, 2022; Zlobina et al., 2006).

Second, the results reaffirm that migrant children’s perceptions of discrimination are not uniformly distributed but are shaped by specific sociodemographic factors. Migrant children from rural–urban migration backgrounds, residing in areas with high proportions of non-local residents, or whose fathers have lower levels of education and occupational status, report significantly higher levels of perceived discrimination. These findings underscore the structural and contextual nature of discrimination and its differential effects within the migrant children population (Spencer et al., 2003b; Xiong et al., 2021).

Third, this study makes a novel contribution by demonstrating that identification with the college matriculation policy—a form of institutional social support—exerts a significant positive influence on migrant children’s urban cultural adaptation. Moreover, the moderating analysis reveals that policy identification attenuates the negative effects of perceived discrimination on all three adaptation dimensions. This finding advances social identity theory (Tajfel & Turner, 1986), which posits that perceived permeability of group boundaries shapes identity strategies and intergroup dynamics. The college matriculation policy enhances boundary permeability by affording migrant children tangible opportunities for social mobility, thereby fostering a more inclusive identity and promoting adaptive outcomes.

Furthermore, this study extends existing scholarship by positioning educational policy as not merely an academic instrument but also as a psychosocial resource that mitigates the detrimental effects of discrimination. In this regard, the findings resonate with prior research emphasizing the protective role of social support in promoting the well-being and adaptation of immigrant and migrant groups (Jasinskaja-Lahti et al., 2006; Spencer et al., 2003a).

In summary, this study offers compelling empirical support for the dual role of the college matriculation policy in enhancing migrant children’s cultural adaptation and buffering against the adverse effects of perceived discrimination. These insights contribute to the theoretical understanding of cultural adaptation processes and have significant practical implications for the design of inclusive educational policies in urban China.

## 6. Implications

This study proposes the following recommendations to further improve migrant children’s cultural adaptation in destination areas:

First, further advance reform of the household registration system; although this system played a stabilizing role in the early years of the People’s Republic, its negative impacts have become increasingly apparent alongside urbanization, restricting migrant children’s rights to sit for secondary and higher education entrance exams and to enjoy equal educational opportunities in host cities. The results show that the registered residence system is the key institutional factor that causes migrant children to face discrimination perception and cultural adaptation problems in their host cities. The registered residence system restricts their eligibility to participate in the college entrance examination and to access equal educational opportunities. The 2014 State Council “Opinions on Further Promoting the Reform of the Household Registration System” has driven college matriculation policy reform and increased migrant children’s chances to take the college entrance exam in their destination cities. Therefore, on one hand, this policy must continue to be implemented to achieve new-type urbanization and equalization of household registration status for urban and rural residents, and on the other hand, local governments and relevant departments should further refine related measures, for example, by incorporating migrant worker residential areas into unified urban planning and by granting them equal citizen rights in medical care, employment, community services, school enrollment, and advancement examinations.

Second, further advance reform of the college entrance admission system. This study found that the current college entrance examination registration conditions (“registered residence registration+school registration”) significantly and negatively impact on migrant children’s perception of discrimination and cultural adaptation. Therefore, the current “household registration+student status” requirement for Gaokao registration should be revised to “residence permit+student status” or “multi-year tax payment certificate+student status.” This change would underscore the importance of student status, effectively curb exam migration, and enable migrant children who have lived and studied long-term in the destination area to sit for out-of-district college entrance examinations. Published out-of-district Gaokao schemes in so-called pioneering breakthrough provinces do not impose a household registration requirement but do require complete high-school student status. In the two most disputed municipalities, Beijing and Shanghai, the policies introduced are largely transitional. For example, Shanghai requires applicants to reach a certain points threshold. Although migrant families may find it difficult to meet the points requirement, it represents an improvement over the household registration criterion.

Third, further secure educational funding for migrant children. The research results indicate that the cultural adaptation of migrant children is influenced by the uneven distribution of educational resources in their destination cities. Therefore, to address the college matriculation policy issue, regions receiving large numbers of migrant children should be granted additional educational funding support and compensation. This approach can alleviate local government financial pressure and allocate more public education resources, thereby better enabling migrant children to receive compulsory education and sit for advancement examinations in destination areas. At the same time, the U.S. “education voucher” model can be referenced: migrant children would carry funding in voucher form to the destination city, and each school that enrolls a migrant child would receive one corresponding education voucher. In addition, drawing on international practice, a provincial-level “Migrant Children Education Fund” could be established. Under this scheme, public schools admitting migrant children would receive an annual per-student subsidy, and through a “government purchase of services” mechanism, migrant children’s right to equal educational opportunity would be guaranteed.

Fourthly, research on the role of social support systems has found that social support has a significant moderating effect on the perception of discrimination and cultural adaptation of migrant children. Therefore, it is recommended to strengthen the construction of the social support system, including the following:

(a) Providing psychological support: Establishing mental health counseling centers in schools and communities to provide psychological support and counseling services for migrant children, helping them cope with the psychological pressure caused by perceived discrimination.
(b) Enhancing community participation: Encouraging migrant children and their families to actively participate in community activities to foster a sense of belonging and identity and reduce the perception of discrimination caused by group isolation.
(c) Promoting policy awareness: Improving the promotion and education of the college entrance examination policy across regions to enhance migrant families’ understanding and sense of policy inclusion, thereby reducing the negative impact of discrimination perception on cultural adaptation.

In summary, the introduction and implementation of the college matriculation policy have, to some extent, compensated for the lack of social capital among migrant children and their families and mitigated the negative impact of perceived discrimination on their urban cultural adaptation. Clearly, the college matriculation policy is an indispensable “good governance” measure. However, for this “good governance” to achieve its intended effect, “good performance” by government departments at all levels, communities, and schools is also required.

## 7. Limitations and Future Research

Though contributions have been made by this study, there are still some limitations. First, the sample size is limited. Sample selection was based on accessibility and convenience, leading to a lack of comprehensiveness and generality in the results. For example, since the participants mainly come from specific cities or regions, the research results may not fully reflect the true situation in other regions or among a wider population. Future research will leverage large-scale Chinese survey platforms such as CEPS and CFPS to embed scales on Chinese education policy identification for wider studies. Second, the experimental hypotheses are not sufficiently developed. This paper focuses on variance analysis of control variables including migration type under the household registration system, father’s education level, and father’s occupation type, without considering the heterogeneity of migrant children across different ages, ethnicities, and social classes. Subsequent studies will further refine the experimental design by incorporating additional potential influencing factors. Third, this paper emphasizes quantitative research methods, resulting in somewhat insufficient accumulation and analysis of qualitative interview data; future work will continue to collect and analyze relevant qualitative materials. Finally, our study’s cross-sectional design limits causal inferences. While we found a significant negative correlation between perceived discrimination and urban cultural adaptation, we could not establish the direction of causality. The lack of longitudinal data also hinders tracking changes in migrant children over time, thus limiting our understanding of the dynamic process of urban cultural adaptation. Future research should consider longitudinal designs to provide stronger evidence for causal relationships.

## Figures and Tables

**Table 1:** Demographic characteristics of the sample.

Variables	*N* (%)	Variables	*N* (%)
**Sex**		**Residence personnel structure**	
Female	892 (62.1)	Many outsiders	95 (6.6)
Male	544 (37.9)	Almost the same	501 (34.9)
**Type of household mobility**		Many locals	840 (58.5)
“Rural-urban” mobility	1144 (79.7)	**Father’s vocational type**	
“Town-town” mobility	292 (20.3)	Unemployed, temporary, or agricultural workers	221 (15.4)
**Father’s level of education**		Ordinary workers, self-employed persons	607 (42.3)
Primary school or below	27 (1.8)	General management or technical personnel	312 (21.7)
Junior high school	564 (39.3)	Middle management or technical personnel	286 (19.9)
Senior high school	868 (56.3)	Senior management or technical personnel, private entrepreneur	10 (0.7)
Undergraduate	37 (2.6)		

Note. *N* = 1436.

**Table 2:** Results of confidence analyses for key variables.

Total Scale	Subscale	Cronbach’s Alpha	
Perceived discrimination		0.938	
College matriculation policy		0.955	
Urban cultural adaptation	Campus culture adaptation	0.956 (total scale)	0.936
	Community culture adaptation		0.920
	Customs and language adaptation		0.923

**Table 3:** Exploratory factor analysis result.

	Perceived Discrimination	College Matriculation Policy	Urban Cultural Adaptation
KMO value	0.957	0.903	0.967
Bartlett test	2895.642 **	3090.116 **	5975.915 **
Number of factors	1	1	3
Cumulative variance explained	61.027%	64%	81.649%

Note: ** indicate *p* < 0.01.

**Table 4:** LR test result.

	Perceived Discrimination	College Matriculation Policy	Urban Cultural Adaptation
Prob > χ^2^	0.000	0.000	0.000
CD	0.971	0.965	0.999
CFI	0.874	0.953	0.809
TLI	0.832	0.943	0.782
RMSEA	0.231	0.100	0.178

**Table 5:** Results of independent sample *t*-test.

Variables and Grouping		Perceived Discrimination	College Matriculation Policy	Campus Culture Adaptation	Community Culture Adaptation	Customs and Language Adaptation
Gender	Male	3.28 ± 0.76	2.95 ± 0.89	3.07 ± 0.92	2.82 ± 0.94	2.85 ± 1.01
	Female	2.86 ± 0.73	3.13 ± 0.87	3.56 ± 0.77	3.22 ± 0.94	3.24 ± 1.00
	T-test	−6.010	−8.343 *	−11.582 **	−8.344	−7.945
Type of household mobility	Rural-urban	3.04 ± 0.74	2.98 ± 0.89	3.05 ± 0.87	2.79 ± 0.93	2.81 ± 0.99
	Urban-urban	2.83 ± 0.74	3.44 ± 0.83	3.90 ± 0.59	3.54 ± 0.82	3.58 ± 0.89
	T-test	−5.168 *	−9.583 **	−19.500 **	−15.044 **	−14.543 **

Note: * *p* < 0.05, ** *p* < 0.01, ±size of the standard deviation.

**Table 6:** Results of one-way analysis of variance.

Variables and Grouping		Perceived Discrimination	College Matriculation Policy	Campus Culture Adaptation	Community Culture Adaptation	Customs and Language Adaptation
Structure of people in the place of residence	(a) More non-locals	3.17 ± 0.79	2.70 ± 0.81	2.55 ± 0.55	2.33 ± 0.71	2.22 ± 0.67
	(b) About the same	3.00 ± 0.70	3.04 ± 0.88	3.21 ± 0.90	2.93 ± 0.95	2.93 ± 0.99
	(c) More local	2.80 ± 0.74	3.53 ± 0.82	3.99 ± 0.48	3.64 ± 0.72	3.79 ± 0.70
	F-test and post hoc comparisons #	31.438 ** a > b > c	121.572 ** c > b > a	549.839 ** c > b > a	317.384 ** c > b > a	456.93 ** c > b > a
Father’s education level	(a) Junior school	3.09 ± 0.91	2.07 ± 0.71	2.88 ± 0.58	2.72 ± 0.73	2.36 ± 0.65
	(b) High school	3.19 ± 0.68	2.63 ± 0.72	2.50 ± 0.81	2.34 ± 0.66	2.37 ± 0.69
	(c) Bachelor’s degree	2.95 ± 0.79	3.04 ± 0.87	3.04 ± 0.90	2.70 ± 1.00	2.73 ± 1.04
	(d) Master’s degree or above	2.91 ± 0.72	3.25 ± 0.92	3.30 ± 0.89	3.33 ± 0.85	3.36 ± 0.94
	F-test and post hoc comparisons #	18.160 ** a > b > c > d	23.092 ** d > c > b > a	117.421 ** d > c > a > b	90.939 ** d > c > a > b	83.407 ** d > c > b > a
Father’s occupation type	(a) Unemployed, temporary workers or agricultural laborers	3.66 ± 0.76	2.87 ± 0.81	2.79 ± 0.63	2.51 ± 0.87	2.47 ± 0.87
	(b) Ordinary workers or self-employed individuals	3.17 ± 0.78	2.78 ± 0.88	2.86 ± 0.85	2.54 ± 0.87	2.51 ± 0.90
	(c) General management personnel and technical personnel	3.01 ± 0.65	3.44 ± 0.87	3.84 ± 0.71	3.54 ± 0.79	3.66 ± 0.83
	(d) Mid-level managers and technical staff	2.82 ± 0.62	3.67 ± 0.59	3.98 ± 0.37	3.76 ± 0.40	3.84 ± 0.51
	(e) Senior management personnel, technical personnel, private business owners	2.70 ± 0.78	3.05 ± 0.98	4.50 ± 0.00	4.50 ± 0.00	4.33 ± 0.17
	F-test and post hoc comparisons #	30.411 ** a > b > c > d > e	84.730 ** d > c > e > a > b	227.844 ** e > d > c > b > a	214.658 ** e > d > c > b > a	238.069 ** e > d > c > b > a

Note: ** *p* < 0.01, ±size of the standard deviation. # full post hoc comparison results can be seen in the Supplementary Materials.

**Table 7:** Correlation analysis results.

	(1)	(2)	(3)	(4)	(5)
(1) College matriculation policy acceptance	1				
(2) Perceived discrimination	−0.382 **	1			
(3) Campus culture adaptation	0.717 **	−0.428 **	1		
(4) Community culture adaptation	0.814 **	−0.372 **	0.428	1	
(5) Customs and language adaptation	0.786 **	−0.387 **	0.362 *	0.348 *	1

Note: * *p* < 0.05, ** *p* < 0.01.

**Table 8:** Test of the moderating effect of college matriculation policy on the relationship between perceived discrimination and campus cultural adaptation.

Variable	Model 1	Model 2	Model 3	Model 4
	Standardized Coefficient	Standardized Coefficient	Standardized Coefficient	Standardized Coefficient
Gender	−0.228 ***	−0.209 ***	−0.129 ***	−0.078 *
Type of household mobility	0.215 ***	0.204 ***	0.145 ***	0.076 *
Structure of people in the place of residence	0.369 ***	0.343 ***	0.254 ***	0.155
Father’s education level	0.171 ***	0.144 ***	0.113 ***	0.070 *
Father’s occupation type	0.220 ***	0.203 ***	0.124 ***	0.115 **
Perceived discrimination		−0.160 ***	−0.097 **	−0.030
College matriculation policy acceptance			0.375 ***	0.470
Interaction term of perceived discrimination and college matriculation policy				0.475
*R* ^2^	0.580	0.601	0.683	0.779
Adjusted *R*^2^	0.572	0.592	0.675	0.772
F-test	73.442 ***	173.881 ***	264.339 ***	319.727 ***

Note: * *p* < 0.05, ** *p* < 0.01, *** *p* < 0.001.

**Table 9:** Test of the moderating effect of college matriculation policy on the relationship between perceived discrimination and community cultural adaptation.

Variable	Model 5	Model 6	Model 7	Model 8
	Standardized Coefficient	Standardized Coefficient	Standardized Coefficient	Standardized Coefficient
Gender	−0.176 ***	−0.159 **	−0.017	−0.003
Type of household mobility	0.142 **	0.131 **	0.028	0.009
Structure of people in the place of residence	0.252 ***	0.228 ***	0.072	0.044
Father’s education level	0.131 *	0.106 *	0.053	0.041
Father’s occupation type	0.313 ***	0.299 ***	0.159 ***	0.156
Perceived discrimination		−0.143 **	−0.032	−0.024
College matriculation policy acceptance			0.661 ***	0.665
Interaction term of perceived discrimination and college matriculation policy				0.132 *
*R* ^2^	0.434	0.451	0.705	0.713
Adjusted *R*^2^	0.423	0.439	0.698	0.704
F-test	40.807 ***	107.805 ***	291.506 ***	361.267

Note: * *p* < 0.05, ** *p* < 0.01, *** *p* < 0.001.

**Table 10:** Test of the moderating effect of college matriculation policy on the relationship between perceived discrimination and customs and language adaptation.

Variable	Model 9	Model 10	Model 7	Model 8
	Standardized Coefficient	Standardized Coefficient	Standardized Coefficient	Standardized Coefficient
Gender	−0.153 **	−0.136 ***	−0.011	0.000
Type of household mobility	0.114 *	0.103 *	0.013	−0.002
Structure of people in the place of residence	0.352 ***	0.328 ***	0.190 ***	0.168
Father’s education level	0.123 *	0.098 *	0.051	0.041
Father’s occupation type	0.283 ***	0.268 ***	0.145 **	0.143 **
Perceived discrimination		−0.146 **	−0.049	−0.022 *
College matriculation policy acceptance			0.581 ***	0.586
Interaction term of perceived discrimination and college matriculation policy				0.104 *
*R* ^2^	0.480	0.498	0.694	0.699
Adjusted *R*^2^	0.470	0.487	0.686	0.690
F-test	43.841 ***	115.094 ***	86.240 ***	368.014 ***

Note: * *p* < 0.05, ** *p* < 0.01, *** *p* < 0.001.

## Data Availability

The data presented in this study are available on reasonable request from the corresponding author. The data are not publicly available due to privacy restrictions.

## Supplementary Materials

The following supporting information can be downloaded at: https://www.mdpi.com/article/10.3390/bs15081136/s1, Table S1. Post hoc verification of the structure of people in the place of residence. Table S2. Post hoc verification of the father’s education level. Table S3. Post hoc verification of the father’s occupation type.
