# Supplementary material for: The Impact of College Matriculation Policies on the Cultural Adaptation of Migrant Children: A Statistical Analysis of Perceived Discrimination in Chinese Cities
Source: Behav Sci (Basel). 2025 Aug 21;15(8):1136. doi: 10.3390/bs15081136 (PMC12382961; doi:10.3390/bs15081136)
Supplement: Supplementary file 1 [file behavsci-15-01136-s001.zip › behavsci-3727777-supplementary.pdf]

Supplementary Materials

Please note that Tukey's HSD test was performed for post-hoc analysis.

Table S1. Post hoc verification of the structure of people in the place of residence

Outcome Variable	Comparison	Mean Difference	P-value
Perceived Discrimination	More non-locals vs. About the same	0.17	< .001**
	More non-locals vs. More locals	0.37	< .001**
	About the same vs. More locals	0.20	< .001**
College Matriculation Policy	More locals vs. About the same	0.49	< .001**
	More locals vs. More non-locals	0.83	< .001**
	About the same vs. More non-locals	0.34	< .001**
Campus Culture Adaptation	More locals vs. About the same	0.78	< .001**
	More locals vs. More non-locals	1.44	< .001**
	About the same vs. More non-locals	0.66	< .001**
Community Culture Adaptation	More locals vs. About the same	0.71	< .001**
	More locals vs. More non-locals	1.31	< .001**
	About the same vs. More non-locals	0.60	< .001**
Customs and Language Adaptation	More locals vs. About the same	0.86	< .001**
	More locals vs. More non-locals	1.57	< .001**
	About the same vs. More non-locals	0.71	< .001**

Table S2. Post hoc verification of the father's education level

Outcome Variable	Comparison	Mean Difference	P-value
Perceived Discrimination	High school vs. Junior school	0.10	.048*
	Bachelor's vs. Junior school	0.14	.007**
	Master's+ vs. Junior school	0.18	< .001**
	High school vs. Bachelor's	0.24	.002**
	High school vs. Master's+	0.28	< .001**
	Bachelor's vs. Master's+	0.04	.612
College Matriculation Policy	Junior school vs. High school	0.56	< .001**
	Junior school vs. Bachelor's	0.97	< .001**
	Junior school vs. Master's+	1.18	< .001**
	High school vs. Bachelor's	0.41	< .001**
	High school vs. Master's+	0.62	< .001**
	Bachelor's vs. Master's+	0.21	.039*
Campus Culture Adaptation	Junior school vs. High school	0.38	< .001**
	Junior school vs. Bachelor's	0.16	.028*
	Junior school vs. Master's+	0.42	< .001**
	High school vs. Bachelor's	0.54	< .001**
	High school vs. Master's+	0.80	< .001**
	Bachelor's vs. Master's+	0.26	< .001**
Community Culture	Junior school vs. High school	0.38	< .001**

Adaptation	Junior school vs. Bachelor’s	0.02	.820
	Junior school vs. Master’s+	0.61	< .001**
	High school vs. Bachelor’s	0.36	< .001**
	High school vs. Master’s+	0.99	< .001**
	Bachelor’s vs. Master’s+	0.63	< .001**
Customs and Language Adaptation	Junior school vs. High school	0.01	.954
	Junior school vs. Bachelor’s	0.37	< .001**
	Junior school vs. Master’s+	1.00	< .001**
	High school vs. Bachelor’s	0.36	< .001**
	High school vs. Master’s+	0.99	< .001**
	Bachelor’s vs. Master’s+	0.63	< .001**

Table S3. Post hoc verification of the father's occupation type

Outcome Variable	Comparison	Mean Difference	P-value
Perceived Discrimination	Unemployed/agricultural vs. Ordinary workers	0.49	< .001**
	Unemployed/agricultural vs. General tech/management	0.65	< .001**
	Unemployed/agricultural vs. Mid-level management	0.84	< .001**
	Unemployed/agricultural vs. Senior/owner	0.96	< .001**
	Ordinary workers vs. General tech/management	0.16	< .001**
	Ordinary workers vs. Mid-level management	0.35	< .001**
	Ordinary workers vs. Senior/owner	0.47	< .001**
	General tech/management vs. Mid-level management	0.19	< .001**
	General tech/management vs. Senior/owner	0.31	< .001**
	Mid-level management vs. Senior/owner	0.12	< .001**
College Matriculation Policy	Mid-level vs. General tech/management	0.23	< .001**
	Mid-level vs. Unemployed/agricultural	0.80	< .001**
	Mid-level vs. Ordinary workers	0.89	< .001**
	General tech/management vs. Unemployed/agricultural	0.57	< .001**
	General tech/management vs. Ordinary workers	0.66	< .001**
	Senior/owner vs. Unemployed/agricultural	0.18	< .001**
	Senior/owner vs. Ordinary workers	0.27	< .001**
	Senior/owner vs. General tech/management	-0.39	< .001**
	Senior/owner vs. Mid-level management	-0.62	< .001**
Campus Culture Adaptation	Senior/owner vs. Mid-level management	0.52	< .001**
	Senior/owner vs. General tech/management	0.66	< .001**
	Senior/owner vs. Ordinary workers	1.64	< .001**
	Senior/owner vs. Unemployed/agricultural	1.71	< .001**
	Mid-level vs. General tech/management	0.14	< .001**
	Mid-level vs. Ordinary workers	1.12	< .001**
	Mid-level vs. Unemployed/agricultural	1.19	< .001**

	General tech/management vs. Ordinary workers	0.98	< .001**
	General tech/management vs. Unemployed/agricultural	1.05	< .001**
	Ordinary workers vs. Unemployed/agricultural	0.07	< .05*
Community Culture Adaptation	Senior/owner vs. Mid-level management	0.74	< .001**
	Senior/owner vs. General tech/management	0.96	< .001**
	Senior/owner vs. Ordinary workers	1.96	< .001**
	Senior/owner vs. Unemployed/agricultural	1.99	< .001**
	Mid-level vs. General tech/management	0.22	< .01**
	Mid-level vs. Ordinary workers	1.22	< .001**
	Mid-level vs. Unemployed/agricultural	1.25	< .001**
	General tech/management vs. Ordinary workers	1.00	< .001**
	General tech/management vs. Unemployed/agricultural	1.03	< .001**
	Ordinary workers vs. Unemployed/agricultural	0.03	n.s.
Customs and Language Adaptation	Senior/owner vs. Mid-level management	0.49	< .001**
	Senior/owner vs. General tech/management	0.67	< .001**
	Senior/owner vs. Ordinary workers	1.82	< .001**
	Senior/owner vs. Unemployed/agricultural	1.86	< .001**
	Mid-level vs. General tech/management	0.18	< .01**
	Mid-level vs. Ordinary workers	1.33	< .001**
	Mid-level vs. Unemployed/agricultural	1.37	< .001**
	General tech/management vs. Ordinary workers	1.15	< .001**
	General tech/management vs. Unemployed/agricultural	1.19	< .001**
	Ordinary workers vs. Unemployed/agricultural	0.04	n.s.
